# 463. Immunogenicity and Safety of mRNA-based SARS-CoV-2 vaccination in Hematopoietic Stem Cell Transplant Recipients: A Systematic Review

**DOI:** 10.1093/ofid/ofad500.533

**Published:** 2023-11-27

**Authors:** Jorge Luis Cardenas-Alvarez, Abel Triana, Juan Uribe, Dan Morgenstern-Kaplan, John M Reynolds, Mohammed Raja, Yoichiro Natori

**Affiliations:** Columbia University, New York, New York; University of Miami/Jackson Memorial Hospital, Miami, Florida; University of Miami/Jackson Memorial Hospital, Miami, Florida; Jackson Memorial Hospital/University of Miami, Miami, Florida; Louis Calder Memorial Library/University of Miami, Miami, Florida; University of Miami Miller School of Medicine/Sylvester Comprehensive Cancer Center, Miami, Florida; University of Miami, Miami Transplant Institute, Jackson Health System, Miami, Florida

## Abstract

**Background:**

Immunization is an effective strategy to decrease hospitalization and mortality related to SARS-CoV-2. Since the introduction of mRNA-based SARS-CoV-2 vaccines (BNT1261b2 and mRNA-1273), several studies have outlined the immunogenicity and safety of these in hematopoietic stem cell transplant (HSCT) recipients. Herein, we describe the first systematic review and meta-analysis with pooled data of available evidence on SARS-CoV-2 vaccination in this patient population.

**Methods:**

We conducted a systematic review and meta-analysis of prospective cohort studies up to May 24 2022 in adult HSCT recipients who were vaccinated with ≥1 doses of either mRNA-based SARS-CoV-2 vaccine. The outcomes measured included: a) vaccine immunogenicity, b) safety, and c) breakthrough SARS-CoV-2 infections. Patients with solid organ transplant or prior documented history of SARS-CoV-2 infection were excluded.

**Results:**

1084 unique studies were identified. After abstracts review, 81 full-text studies were assessed by two independent reviewers. Twenty-nine studies were included. A total of 1738 patients were analyzed, out of which 1478 received allogeneic-HCST (Allo-HSCT) and 260 autologous-HSCT (Auto-HSCT). Median IgG positivity after at least 1 dose of vaccination (among studies) was 77.3% (range: 0-100%) among Allo-HSCT and 91.7% (range: 60-100%) among Auto-HSCT. Cellular immunity was reported in 5 studies, describing IFN-gamma and/or lymphocyte response. Median positivity was 42% (range: 24-78%) among Allo-HSCT. Pooled incidence of adverse events reported among all HSCT recipients was 38.72%. The most common side effect was injection-site pain. No grade 5 adverse events were described. De-novo or exacerbation of graft versus host disease was reported in the median of 2.28% (range: 0-6.6%) of Allo-HSCT recipients. Breakthrough COVID-19 infections were reported in both Allo-HSCT and Auto-HSCT in 1/479 (0.2%) and 2/254 (1.29%), respectively.

**Figure 1.**
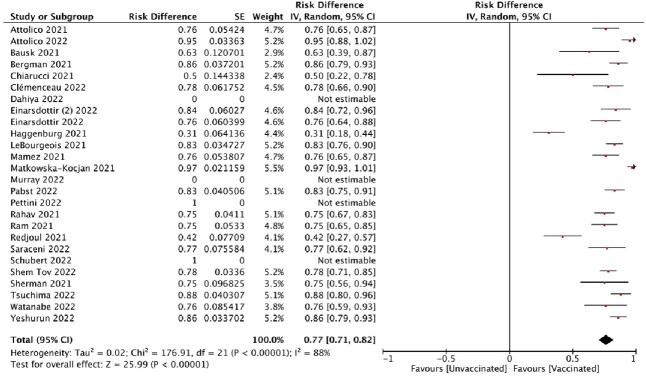
Pooled incidence of antibody positivity after one or more doses of mRNA-based SARS-CoV-2 vaccination in HSCT recipients

**Conclusion:**

mRNA-based SARS-CoV-2 vaccination was immunogenic in HSCT recipients, particularly among Auto-HSCT recipients. One or more doses of mRNA-based vaccine elicited a greater humoral response, compared to that of cellular immunity. Adverse events were common, but often were mild. Breakthrough infections are rare after ≥ 1 doses.

**Disclosures:**

**John M. Reynolds, MLIS**, Pfizer Inc: Stocks/Bonds

